# Treatment burden in multimorbidity: an integrative review

**DOI:** 10.1186/s12875-024-02586-z

**Published:** 2024-09-28

**Authors:** Ji Eun Lee, Jihyang Lee, Rooheui Shin, Oonjee Oh, Kyoung Suk Lee

**Affiliations:** 1 College of Nursing, Seoul National University, 103 Daehak-ro, Jongno-gu, Seoul, 03080 South Korea; 2 Center for World-leading Human-care Nurse Leaders for the Future by Brain Korea 21 (BK 21) four project, College of Nursing, Seoul National University, Seoul, South Korea; 3 School of Nursing, University of Pennsylvania, Philadelphia, PA USA; 4 Research Institute of Nursing Science, College of Nursing, Seoul National University, Seoul, South Korea

**Keywords:** Adherence, Comorbidity, Healthcare tasks, Multimorbidity, Treatment burden

## Abstract

**Background:**

People living with multimorbidity experience increased treatment burden, which can result in poor health outcomes. Despite previous efforts to grasp the concept of treatment burden, the treatment burden of people living with multimorbidity has not been thoroughly explored, which may limit our understanding of treatment burden in this population. This study aimed to identify the components, contributing factors, and health outcomes of treatment burden in people with multiple diseases to develop an integrated map of treatment burden experienced by people living with multimorbidity. The second aim of this study is to identify the treatment burden instruments used to evaluate people living with multimorbidity and assess the comprehensiveness of the instruments.

**Methods:**

This integrative review was conducted using the electronic databases MEDLINE, EMBASE, CINAHL, and reference lists of articles through May 2023. All empirical studies published in English were included if they explored treatment burden among adult people living with multimorbidity. Data extraction using a predetermined template was performed.

**Results:**

Thirty studies were included in this review. Treatment burden consisted of four healthcare tasks and the social, emotional, and financial impacts that these tasks imposed on people living with multimorbidity. The context of multimorbidity, individual’s circumstances, and how available internal and external resources affected treatment burden. We explored that an increase in treatment burden resulted in non-adherence to treatment, disease progression, poor health status and quality of life, and caregiver burden. Three instruments were used to measure treatment burden in living with multimorbidity. The levels of comprehensiveness of the instruments regarding healthcare tasks and impacts varied. However, none of the items addressed the healthcare task of ongoing prioritization of the tasks.

**Conclusions:**

We developed an integrated map illustrating the relationships between treatment burden, the context of multimorbidity, people’s resources, and the health outcomes. None of the existing measures included an item asking about the ongoing process of setting priorities among the various healthcare tasks, which highlights the need for improved measures. Our findings provide a deeper understanding of treatment burden in multimorbidity, but more research for refinement is needed. Future studies are also needed to develop strategies to comprehensively capture both the healthcare tasks and impacts for people living with multimorbidity and to decrease treatment burden using a holistic approach to improve relevant outcomes.

**Trial registration:**

DOI: https://doi.org/10.17605/OSF.IO/UF46V

**Supplementary Information:**

The online version contains supplementary material available at 10.1186/s12875-024-02586-z.

## Background

Multimorbidity, the co-existence of two or more chronic diseases, is a major global health issue affecting over one-third of the population [1, 2]. People living with multimorbidity encounter unique challenges of simultaneously managing multiple conditions, such as managing polypharmacy, and conflicting treatment regimens, while also coping with altered physical and mental function [3–6]. An ineffective and fragmented healthcare system that focuses on a single disease can add challenges to understanding and navigating healthcare tasks, which, in turn, can exacerbate people’s treatment burden [7–9]. Recognizing that the healthcare system contributes to people’s treatment burden, May and colleagues proposed the concept of minimally disruptive medicine [10]. This approach emphasizes coordinated and patient-centered collaborative care services designed to reduce people’s treatment burden. Minimally disruptive medicine helps to streamline the care process, making treatment of health conditions less burdensome and more manageable for people’s daily lives [10, 11].

Treatment burden refers to patients’ workload in treating and managing chronic health conditions and the combined impact on their well-being [12]. Treatment burden is recognized as an important patient-reported outcome in people living with multimorbidity [13]. Considerable research has focused on understanding the attributes and characteristics of treatment burden in multimorbidity [14–17]. Two groups of investigators developed the conceptual framework or taxonomy of treatment burden in multimorbidity [18, 19]. Despite the substantial scholarly progress in understanding the treatment burden in multimorbidity, a significant knowledge gap remains for three reasons. First, existing studies have identified the contributing factors and components of treatment burden but have not addressed health outcomes resulting from treatment burden. For example, the two research groups included factors exacerbating treatment burden, elements of work or tasks people living with multimorbidity must perform, and the impacts of the tasks on patients’ well-being (e.g., emotional impact, social activity limitations) [18, 19]. Second, the elements of treatment burden have been identified from a limited number of empirical studies and they have not specifically examined people living with multimorbidity. For example, Tran and colleagues recruited a large number of participants from three Western countries [19]. However, their suggested taxonomy was developed based on a single quantitative study in which the sample was not limited to people living with multimorbidity. Third, while review studies have synthesized treatment burden [17, 20, 21], they have primarily focused on people with chronic conditions [20, 21] and have included only qualitative [20] or quantitative studies [17]. Thus, the unique aspects of the treatment burden experienced by people living with multimorbidity have not been fully elucidated in existing conceptual framework and taxonomy. Due to this knowledge gap, the current measures for treatment burden may not capture the distinct aspects of treatment burden experienced by people living with multimorbidity [22]. Therefore, it is important to also evaluate the contents of the instruments that have been used to measure treatment burden in people living with multimorbidity.

The purpose of this integrative review is to gain a comprehensive understanding of the treatment burden experienced by people living with multimorbidity by synthesizing the empirical literature on the treatment burden of people living with multimorbidity, and evaluate the treatment burden measures. The specific aims are 1) to identify the components of treatment burden, contributing factors, and health outcomes of treatment burden as revealed in the literature and 2) to evaluate the comprehensiveness of the instruments that have been used to assess treatment burden in people living with multiple conditions.

## Methods

This review was registered in the Open Science Framework on September 5, 2022 (https://doi.org/10.17605/OSF.IO/UF46V) [23]. To provide a more comprehensive understanding of treatment burden in people living with multiple conditions, we made two key modifications to our original protocol. First, we extended the literature search to include all available years rather than limiting it to the last 10 years. Second, we changed our review methodology from a scoping review to an integrative review, which allows for the inclusion of diverse research methodologies such as quantitative and qualitative studies. We followed the steps outlined by Whittemore and Knafl for the integrative review process: problem identification, literature search and selection, data evaluation, data analysis and presentation [24].

### Search strategy

A systematic search was conducted using three electronic databases: MEDLINE, EMBASE, and CINAHL. The search strategy involved the use of MeSH terms, EMTREE, and/or free text keywords such as "multimorbidity," "comorbidity," "burden," "workload," and other relevant keywords related to treatment burden and specific domains suggested from a previous study describing treatment burden in chronic conditions [25] such as "time," "travel," "financial," and "healthcare." After selecting the included articles, the references were manually searched for additional relevant studies. The search was limited to articles published in English and the year of publication up to May 2023. We consulted with a medical librarian on the search process. Supplementary file 1 lists the MEDLINE search queries.

### Study selection

The studies were selected based on the following inclusion criteria: 1) targeting adults over 19 years with at least two chronic conditions; 2) studies describing any aspects of treatment burden and/or related factors (contributing factors or health outcomes) from perspectives of people living with multimorbidity, and 3) published in English up to May 2023. In our review, chronic disease was defined as a long-term, incurable condition requiring ongoing care [26], and treatment burden as the healthcare workload and its impact on patient well-being [14]. Studies were excluded based on the following criteria: 1) studies measuring the treatment burden of specific conditions (e.g., Diabetic Treatment Burden Questionnaire) [27], 2) studies describing treatment burden from the perspectives of samples other than people living with multimorbidity (e.g., caregivers, healthcare professionals), or 3) non-empirical studies such as review articles. Among the eligible studies, an additional inclusion criterion was applied to analyze the comprehensiveness of the contents of the treatment burden instruments such as studies reporting on the psychometric properties of the measures.

### Data abstraction

All records were collected into a single EndNote library file to delete duplicates, and the remaining records then were exported to an Excel sheet with essential information for screening. Two authors independently screened the titles and abstracts, and then read the full texts of studies based on the eligibility criteria. Any discrepancies were discussed, and a third author resolved disagreements between the authors.

### Quality assessment

Study quality was assessed using the Mixed Methods Appraisal Tool (MMAT) [28, 29]. MMAT is a versatile tool that can be applied across a variety of study designs, including quantitative, qualitative, and mixed methods studies. Each study was evaluated as “yes,” “no,” or “can’t tell” based on five criteria. “Can’t tell” means that appropriate information was not reported or the information was unclear. The ratings for the criteria were presented without calculating the overall score as recommended [30]. Two authors independently evaluated one study and discussed discrepancies to reach a consensus. The evaluation scores for each study are presented in Supplementary file 2.

### Data synthesis

We conducted data analysis following the four steps suggested by Whittemore and Knafl. In the first stage of data reduction, we abstracted the data from the primary sources by organizing studies into groups based on different methodologies (quantitative and qualitative) and predetermined factors (i.e., treatment burden, contributing factors, and health outcomes). The five authors independently extracted data from the full text of each article using a predetermined data extraction template (see Supplementary file 3). The development of the initial template was guided by the aims of our review and then the template was refined through several rounds of discussion among the five authors. We also conducted pilot testing to ensure that we captured all of the necessary information. The extracted data were cross-checked independently by two authors. Any unclear information in the original paper was clarified by contacting the original author(s) of the paper.

In the second stage, displaying the data, we presented the extracted data through matrices and charts. The third step, data comparison, involved an iterative examination of the data displays to identify patterns, themes, or relationships from both quantitative and qualitative data. The key outcomes of the quantitative studies were summarized in a table format, which included inferential statistics (e.g., standardized and unstandardized coefficients with a 95% confidence interval). Results from the multivariate regression analyses were included unless univariate analysis results were only available. We determined the significance by considering a *p*-value threshold of 0.05 and a 95% confidence intervals.

Qualitative data were analyzed by extracting the segments of results that were related to our review aims. These extracted segments were grouped into categories identified during the quantitative data synthesis. The results from both the quantitative and qualitative data were integrated using matrices (Tables 3 and 4), to help identify common patterns and relationships across both types of data. Similarly, items from the instruments measuring treatment burden and the segments of the qualitative results that were relevant to the attributes to treatment burden were displayed side-by-side to compare the data (Table 2). Finally, in the fourth step, conclusion drawing, we developed an integrated map of the treatment burden of multimorbidity based on the previous step. This map provides a comprehensive visual representation of how different factors and outcomes related to treatment burden are interconnected (Fig. 2).

## Results

### Search results

**Fig. 1:**
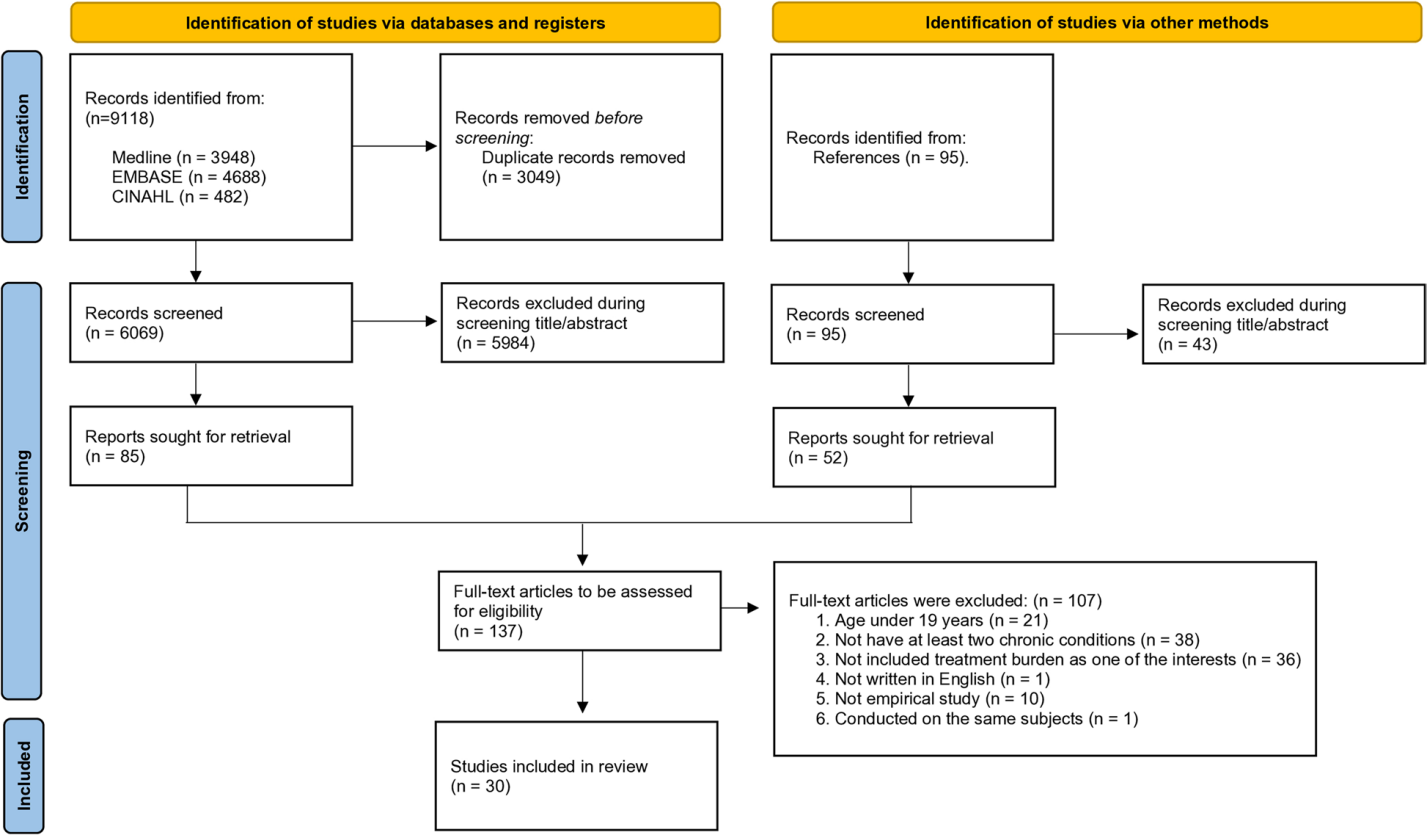
PRISMA flowchart illustrating the systematic reviews

The initial database search resulted in 9118 articles, of which 6069 remained after duplicates were eliminated (Fig. 1). An additional 95 articles were included from the reference lists for screening. After screening the titles and abstracts, 137 full text articles were assessed for eligibility. As a result, 30 studies were included in this integrative review. Of the 30 studies, nine were qualitative studies and 21 were quantitative studies.

### Characteristics of included studies

Of the 30 studies in our sample, 23 targeted people living with multimorbidity. The overall average across the 11 studies reporting the mean number of multimorbidity yielded a mean of 5.38 (SD 2.25). Among the ten studies that reported the median number of diseases, the median ranged from three [12, 31] to five [32–37] (Table 1). The most common inclusion criterion of multimorbidity was having at least two chronic conditions, whereas some studies included people with at least three or four conditions with or without additional criteria (e.g., the number of medications) [38–44]. The remaining seven studies targeted people with index chronic conditions and co-morbidity [5, 6, 9, 45–48]. The most prevalent index conditions in these seven studies were hypertension and/or type 2 diabetes (*n* = 3). Researchers have collected a list of chronic conditions based on medical record reviews (*n* = 20) or self-report (*n* = 5) [37, 38, 49–51] or both (*n* = 3) [6, 40, 41] although two studies did not indicate how they collected this information [5, 9] (Table 1).

**Table 1:** Characteristics of included study (*N* = 30)

Authors (Year)/Country	Study aims	Number of chronic conditions	Data collection for chronic conditions	Instrument to measure treatment burden	Participants	Analytic approach or study design
**Qualitative studies (*n* = 9)**						
Van Pinxteren (2023) /South Africa [9]	To explore and comprehend the workload and capacity related to self-management among multimorbid patient living in precarious circumstances in urban and rural South Africa	- Mean number of conditions: 2.6	NR	NA	- *N* = 30 (70% female) - Mean age, 53.5 years - 100% None-White - Education: NR	Inductive and deductive qualitative thematic analysis
Corbett (2022) /United Kingdom [5]	(1) To identify how older people with MM manage health after they have completed cancer treatment (2) To explore factors impacting workload and capacity for self-care based on the CCM	- Mean number of conditions: 5.3	NR	NA	- *N* = 8 (75% female) - Mean age, 79 years - None-White: NR - 50% below secondary education	Framework analysis
Hardman (2021) /Australia [4]	To investigate, how the demands of MM impact burden and capacity in a low-income population with MM as defined by the CCM	- Mean number of conditions: 7	Self-report	NA	- *N* = 13 (46% female) - Mean age, 61 years - None-White: NR - Education: NR	Framework analysis (based on phenomenological methodology)
Morgan (2019) /Ghana [52]	To examine the perceptions and experiences of women with MM in the region of Ghana, especially how women describe their chronic conditions and health needs and how the health system responds to them, based on the CCM	- Mean number of conditions: 2.7	Medical record	NA	- *N* = 20 (100% female) - Mean age, 54.8 years - None-White: 100% - 75% below secondary education	Thematic analysis
Matima (2018) /South Africa [8]	To explore the workload and capacity of patient with the human immunodeficiency virus and type 2 diabetes comorbidity in the South African context, based on the CCM	- Mean number of conditions: NR	Medical record	NA	- *N* = 10 (50% female) - Mean age, 46.9 years - None-White: 100% - 10% below secondary education	Thematic content analysis
Ørtenblad (2018) /Denmark [38]	To identify the interface of MM, everyday life, and TB in a Danish context	- Mean number of conditions: 4.7	Self-report	NA	- *N* = 10 (50% female) - Mean age, 51.4 years - None-White: NR - Education: unclear	Inductive analytical approach (based on phenomenology and ethnographic methodology)
Van Merode (2018) /Netherlands, Belgium [53]	To investigate the experiences of patient with MM focused on TB	- Mean number of conditions: 3.1	Medical record	NA	- *N* = 22 (68.2% female) - Mean age, 70.9 years - None-White: NR - Education: NR	Thematic content analysis
Duguay (2014) /Canada [39]	To outline the fundamental structure of MM adults’ experience	- Mean number of conditions: 7	Medical record	NA	- *N* = 11 (36% female) - Mean age, 58.1 years - None-White: NR - 45% below secondary education	Inductive analytical approach (based on phenomenology)
Fix (2014) /United States [6]	To comprehend barriers to hypertension self-care in people with hypertension and comorbidities	- Mean number of conditions: NR	Medical record and self-report	NA	- *N* = 48 (10.4% female) - Mean age, 60 years - None-White: NR - 10.4% below high school	Thematic analysis (based on grounded theory
**Observational studies (*n* = 12)**						
Hounkpatin (2022) /United Kingdom [40]	(1) To measure TB change, and identify associated factors (2) To analyze a revised single-item measure for high TB in older adults with MM	- Mean number of conditions: NR	Medical record and self-report	MTBQ, single-item TB tool	- *N* = 300 (56.8% female) - Mean age, 74.5 years - 0.3% None-White - 40.5% below secondary education	Longitudinal design
Eton (2022) /United States [36]	To figure out whether there are different longitudinal patterns of TB in people living with MM and explore predictors	- Median number of conditions: 5	Medical record	PETS version 2.0	- *N* = 396 (62.9% female) - Median age, 63 years - 17.2% None-White - 22.7% below secondary education	Longitudinal design
El-Nagar (2021) /Egypt [51]	To investigate the association between health literacy and TB among patient with MM	- Mean number of conditions: 4.4	Self-report	MTBQ	- *N* = 480 (60% female) - Mean age, 53.1 years - None-White: NR - Education: unclear	Cross-sectional design
Morris (2021) [41] /United Kingdom	(1) To assess the extent of and associations with high TB among older adults with MM (2) To examine the performance of a novel single-item TB measure	- Mean number of conditions: NR	Medical record and self-report	MTBQ, Single-item TB tool	- *N* = 835 (54.6% female) - Mean age, 75 years - 0.8% None-White - Education: NR	Cross-sectional design
Siddiqui (2021) /United States [37]	To better understand how TB presents at the end of life	- Median number of conditions: 5	Self-report	Four items from NHATS	- *N* = 238 (53.8% female) - Mean age, NR - 13.7% None-White - 32.8% below high school education	Cross-sectional design
Hu (2021) /China [45]	To provide insights into the process from the perspective of healthcare needs, patient experiences, and TB	- Mean number of conditions: NR	Medical record	TBQ (Mandarin Chinese version)	- *N* = 2160 (56% female) - Mean age, 61.4 years - None-White: 100% - 55.3% below secondary education	Cross-sectional design
Schreiner (2020) /United States [54]	To assess TB among adults with MM who are transitioning from a skilled nursing facility to home	- Mean number of conditions: 4.3	Medical record	TBQ	- *N* = 74 (74.3% female) - Mean age, 75.4 years - 8.9% None-White - 21.6% below high school	Longitudinal design
Aschmann (2019) /United States [46]	(1) To use best–worst scaling to elicit preferences about patient-important outcomes related to hypertension in people with MM (2) To explore if preferences were associated with patient characteristics	- Mean number of conditions: NR (mean total Quan score: 6.1)	Medical record	NR	- *N* = 217 (49.8% female) - Mean age, 74.5 years - 11.1% None-White - Education: NR	Cross-sectional design
Herzig (2019) /Switzerland [42]	To identify factors related to how patient with MM perceive TB and compare them to general practitioners’ assessment factors	- Mean number of conditions: 7.2	Medical record	TBQ (German version)	- *N* = 888 (51.8% female) - Mean age, 72.9 years - None-White: NR - 22% below secondary education	Cross-sectional design
Eton (2019) /United States [35]	(1) To examine risk factors for poor health-related quality of life in multi-morbid adult cancer survivors (2) To identify if perceived treatment and self-management burdens mediate these relationships	- Median number of conditions: 5	Medical record	PETS	- *N* = 91 (59% female) - Median age, 65 years - 17.6% None-White - 26% below high school	Longitudinal design
Song (2019) /United States [48]	(1) To solicit social networks for self-care and care coordination in patient on dialysis therapy (2) To analyze the variation of network characteristics, (3) To investigate the link between network characteristics and perceived TB	- Mean number of conditions: NR	Medical record	PETS	- *N* = 20 (50% female) - Mean age, 53.4 years - 95% None-White - 30% below high school	Cross-sectional design
Eton (2017) /United States [31]	(1) To determine the associations between healthcare provider relational quality and self-care and psychosocial outcomes in adults with MM (2) To identify if specific indicators of healthcare provider relational quality are more strongly linked to self-care and psychosocial outcomes	- Median number of conditions: 3	Medical record	PETS	- *N* = 332 (56% female) - Mean age, 65.9 years - 27% None-White - 7% below high school	Cross-sectional design
**Interventional studies (*n* = 2)**						
McCarthy (2022) /Ireland [44]	To explore the effect of general practitioner-delivered, personalized medication review in reducing polypharmacy and potential inappropriate prescriptions among elderly with MM in primary care	- Median number of conditions: NR	Medical record	MTBQ	- *N* = 404 (57.2% female) - Mean age, 76.5 years - None-White: NR - 45% below high school	Cluster randomized controlled trial
Tinetti (2019) /United States [43]	(1) To analyze the relationship between patient priority care participation and patients' perception of treatment effectiveness and burden compared to usual care (2) To compare ambulatory healthcare changes in patient receiving priority care participation and usual care	- Median number of conditions: (Intervention: 4.0, control: 3.82)	Medical record	TBQ	- *N* = 366 (64.2% female) - Mean age, 76.7 years - 4.4% None-White - 7.4% below high school	Non-randomized clinical trial
**Studies examining psychometric properties (*n* = 7)**						
Schulze (2022) /Germany [55]	(1) To translate and culturally adapt the MTBQ to German (2) To confirm the adapted version in older adults with MM (3) To analyze the relationship between TB scores and sociodemographic characteristics as well as other patient-reported health measures	- Mean number of conditions: 9.8	Medical record	MTBQ (German version)	- *N* = 344 (55.2% female) - Mean age, 77.5 years - None-White NR - 56.4% below secondary education	Cross-sectional design
Lee (2020) /United States [34]	To analyze the factor structure and differential item functioning of the Patient Experience with Treatment and Self-management version 2.0 (PETS version 2.0)	- Median number of conditions: 5	Medical record	PETS version 2.0	- *N* = 439 (62% female) - Mean age, 60.3 years - 19% None-White - 24% below high school	Cross-sectional design (Test–retest, median retest interval = 18 days)
Dou (2020) /China [56]	To translate and culturally adapt the Multimorbidity Treatment Burden Questionnaire (MTBQ) into Chinese and assess the questionnaire’s psychometric properties	- Mean number of conditions: NR	Medical record	MTBQ (Chinese version)	- *N* = 156 (45.5% female) - Mean age, 73.5 years - None-White: NR - 25% below secondary education	Cross-sectional design (Test–retest, interval = 14 days)
Eton (2020) /United States [33]	(1) To evaluate known-groups validity and responsiveness to change of PETS version 2.0 scales prospectively in patient with MM (2) To determine the usefulness of two newly developed PETS index scores	- Median number of conditions: 5	Medical record	PETS version 2.0	- *N* = 365 (64% female) - Mean age, 62.1 years - 19% None-White - 23% below high school	Longitudinal design (12 months)
Eton (2020) /United States [32]	To develop a concise version of the PETS, based on the longer form and tailored specifically for measuring the quality of person-centered care	- Median number of conditions: 5	Medical record	PETS short version	- *N* = 400 (50% female) - Mean age, 57.9 years - 52.3% None-White - 43% below college educated	Longitudinal design (6–12 months)
Chin (2019) /China [50]	(1) To translate and culturally adapt the TBQ from English to Chinese for use in Hong Kong (2) To explore the psychometric properties of the adapted TBQ among a sample of Chinese primary care	- Median number of conditions: 4	Self-report	TBQ	- *N* = 200 (55% female) - Median age, 62 years - None-White: NR - Education: NR	Cross-sectional design (Test–retest, interval = 14 days)
Eton (2017) /United States [12]	To establish and validate a new comprehensive patient-reported measure of TB, known as the PETS	- Median number of conditions: 3	Medical record	PETS	- *N* = 332 (56% female) - Mean age, 65.9 years - 20% None-White - 7% below high school	Cross-sectional design

*CCM* Cumulative Complexity Model, *MM* Multimorbidity, *MTBQ* Multimorbidity Treatment Burden Questionnaire, *PETS* Patient Experience with Treatment burden and Self-management, *TB* Treatment Burden, *TBQ* Treatment Burden Questionnaire

Treatment burden was measured based on three instruments and their variations: the Patient Experience with Treatment burden and Self-management (PETS) and its variations (*n* = 8) [12, 31–36, 48]; the Treatment Burden Questionnaire (TBQ) (*n* = 5) [42, 43, 45, 50, 54]; and the Multimorbidity Treatment Burden Questionnaire (MTBQ) (*n* = 4) [44, 51, 55, 56], and the MTBQ with a single-item (*n* = 2) [40, 41] and a four-item measure (*n* = 1) [37].

### Methodological quality

Nine qualitative studies met all five quality assessment criteria. In the 19 quantitative descriptive studies, one study (5.3%) met only one criterion [54], two studies (10.5%) met two criteria [48, 51], and the rest (84.2%) met three to four criteria. The most unmet criterion in quantitative descriptive studies (68.4%) was related to the representativeness of the samples. One quantitative randomized controlled trial did not meet one criterion related to adherence to intervention, as more than 20% of participants did not receive a medication review intervention [44]. One quantitative non-randomized study met four criteria, but the authors did not state whether participants were exposed to the intervention as planned [43].

### Components of treatment burden

**Fig. 2:**
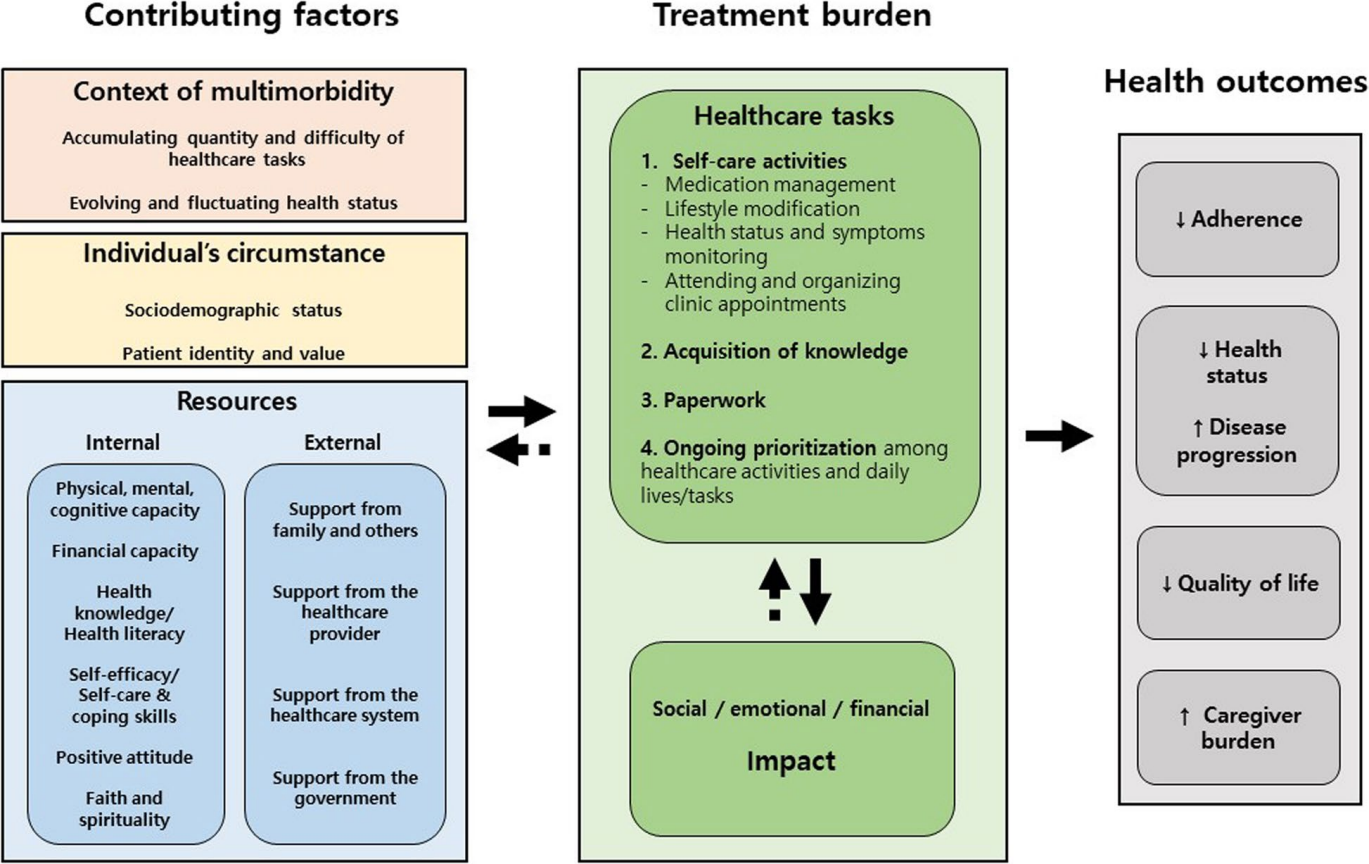
Integrated map of treatment burden in multimorbidity. The dotted line refers to a small number of studies indicating the relationship, implying the scarcity of evidence

The results of the studies included in this review indicated that treatment burden consisted of several healthcare tasks that people living with multimorbidity are asked to perform to manage their health conditions and the impacts of those healthcare tasks on their lives (Fig. 2). Healthcare tasks was interconnected with impacts [4, 5, 8, 9, 38, 39, 52, 53], and two studies indicated that impacts affected healthcare tasks [36, 38].

#### Healthcare tasks

As shown in Table 2, people living with multimorbidity invest time, money, and efforts to engage in four categories of healthcare tasks: self-care activities, knowledge acquisition, paperwork, and ongoing prioritization. The self-care activities category was the most frequently reported across the studies that explicitly mentioned these activities [4–6, 8, 9, 39, 41, 42, 46, 53]. This category included organizing and remembering the medication schedule, and taking multiple medications as directed [4–6, 8, 9, 39, 41, 42, 46, 53]. In addition, people with multimorbidity reported challenges organizing and attending multiple medical appointments including the inconvenience of making transportation arrangements and traveling to multiple clinics on different dates at different locations [4, 5, 8, 9, 38, 39, 52, 53].

**Table 2:** The components of treatment burden identified in empirical studies and instrument of treatment burden

**Treatment burden components**	**Empirical studies (*n* = 8)**	**Instruments** (Number of items, %)		
	**Contents**	**PETS version 2.0** **(60 items)**	**TBQ** **(15 items)**	**MTBQ** **(13 items)**
**Healthcare tasks**		**40 (66.7%)**	**11 (73.4%)**	**10 (77%)**
Self-care activities	1. Medication management (e.g., scheduling and organizing medications) (*n* = 8)	9 (15.0%)	4 (26.7%)	3 (23.1%)
	2. Lifestyle modifications and other activities to manage health conditions (*n* = 7)	9 (15.0%)	2 (13.3%)	1 (7.7%)
	3. Health status and symptoms monitoring (*n* = 3)	2 (3.3%)	1 (6.7%)	1 (7.7%)
	4. Organizing, coordinating, and attending multiple appointments (*n* = 8)	11 (18.3%)	3 (20.0%)	4 (30.8%)^**^
Knowledge acquisition	1. Learn about conditions and treatment (*n* = 1)	7 (11.7%)	0 (0.0%)	1 (7.7%)
Paperwork	1. Doing reimbursement progress (*n* = 1)	1 (1.7%)	1 (6.7%)	0 (0.0%)
	2. Keeping healthcare records (*n* = 1)	1 (1.7%)		0 (0.0%)
Ongoing prioritization	1. Constant prioritization between healthcare tasks and daily lives (e.g., family and work life or/and between healthcare tasks) (*n* = 5)	0 (0.0%)	0 (0.0%)	0 (0.0%)
	2. Prioritization between healthcare tasks (*n* = 2)	0 (0.0%)	0 (0.0%)	0 (0.0%)
**Impacts**		**19 (31.7%)**	**3 (20.1%)**	**2 (15.4%)**
Social impact	1. Role limitations (e.g., threat of being unemployed) (*n* = 2)	2 (3.3%)	0 (0.0%)	0 (0.0%)
	2. Social activity limitations and worsening social relationship (*n* = 5)	7 (11.7%)	0 (0.0%)	0 (0.0%)
	3. Being dependent on others (*n* = 6)	1 (1.7%)	1 (6.7%)	1 (7.7%)
Emotional impact	1. Emotional status (e.g., feeling stressed, exhausted) (*n* = 5)	5 (8.3%)	1 (6.7%)	0 (0.0%)
Financial impact	1. Financial instability (*n* = 2)	4 (6.7%)	1 (6.7%)	1 (7.7%)

*MTBQ *Multimorbidity Treatment Burden Questionnaire, *PETS* Patient Experience with Treatment and Self-Management, *TBQ* Treatment Burden Questionnaire. ‘n’ indicated the number of articles

^**^The final German version of the MTBQ included three items relevant to organizing, coordinating, and attending multiple appointments as opposed to four items in the original version of the MTBQ. The sum of items on each of the three instruments differed from the total number of items because they included items indicating resources, not treatment burden

Another challenge was that people living with multimorbidity spent time and efforts to understand their health conditions, including seeking information from various sources (e.g., websites) and assessing their personal experience [4, 9]. Some people described difficulties obtaining comprehensive information across their multiple diseases [8, 9]. Paperwork was an additional task people performed to reimburse medical costs and maintain their medical records for efficient communication with clinicians [5, 53].

People living with multimorbidity described that they spent a substantial amount of time and efforts evaluating the significance of healthcare tasks in their current situations compared to their other life demands or values (e.g., work and family life) and contemplating the potential impacts of their choices [5, 38, 39, 52, 53]. People also described their efforts to decide what action to take when faced with treatment regimens that seemed incompatible [5, 6]. This prioritization was not static but constant as their situations and values changed over time [5, 38, 39]. For example, one participant reported that she usually placed a high priority on her health condition over her life demands. However, she sometimes chose her social life over her health conditions, although she anticipated negative consequences on her health as a result [38].

#### Impact

Healthcare tasks impacted various aspects of people’s lives, particularly their social, emotional, and financial aspects (Table 2 and Fig. 2) [5, 8, 38, 39, 53]. Asking for help from others, particularly financial support for treatment, made people living with multimorbidity dependent on others, which affected their sense of autonomy [5, 8, 9, 38, 52, 53]. People also expressed negative feelings such as anger, frustration, and a sense of worthlessness when they felt that they did not have control over managing their health conditions. This sense of loss of control was exacerbated by overwhelming demands of healthcare tasks, which posed threats to their well-being (e.g., insecurity maintaining jobs, losing time for leisure) [8, 9, 38, 39, 53]. However, the emotional impact of healthcare tasks was not entirely negative. For instance, in the study by Duguay and colleagues where people living with at least four chronic conditions were recruited in family medicine clinics, people who faithfully adhered to prescribed tasks such as medication and exercise experienced a sense of being healthy [39]. Medical costs to manage health (e.g., purchasing healthy foods and medications and transportation costs) impacted people's financial status. Many people had to rely on their savings or financial support from their families to cover these costs [9, 49].

### Contributing factors that affect treatment burden

The included studies (*n* = 24) indicated that when people had multiple chronic conditions (i.e., the context of multimorbidity), their circumstances and available resources (i.e., internal and external resources) affected their treatment burden (Fig. 2).

#### Context of multimorbidity

Findings from the included studies indicated that healthcare tasks and the impacts on the well-being of people with multimorbidity were complicated due to the management and nature of multimorbidity including the accumulating quantity and difficulty of healthcare tasks and the evolving and fluctuating health status from multiple conditions (Fig. 2). The studies found that having multiple chronic conditions tended to increase treatment burden [5, 32, 33, 40–42, 51, 55, 57], possibly due to the increased number of healthcare tasks, which could also contribute to an increase in the complexity of the healthcare tasks [6, 8, 38, 39, 49, 52, 53]. For example, participants mentioned that taking multiple medications as directed for their various conditions was significant work. It also increased their vigilance to potential interactions between chronic conditions and/or between therapeutic regimens across chronic conditions (e.g., side effects due to medication interactions) and increased their dependency on their family [38, 49]. When people living with multimorbidity perceived that their healthcare tasks were interdependent or incompatible, the difficulty of undertaking these healthcare tasks was amplified [5, 6, 38, 39, 49]. The addition of a new diagnosis or a change in their health status also forced them to integrate their additional healthcare tasks into their existing routines. Duguay and colleagues described this burden as "a wheel that turns" due to the evolving and fluctuating nature of multiple conditions [39]. The dynamic nature of the multiple conditions also contributed to the emotional status of people with multimorbidity, such as feeling that their health trajectory was unpredictable [5, 39, 49].

#### Circumstance-related factors of people with multimorbidity

In 16 studies, a variety of circumstance-related factors were investigated or described in relation to treatment burden (Table 3). Frequently mentioned circumstance-related factors included socio-demographic factors such as place of residence, employment status, identity, and the value of life of people with multimorbidity.

**Table 3:** Contributing factors that affect treatment burden: Circumstance-related factors of people with multimorbidity

Circumstance-related factors	Worsening treatment burden		Reducing treatment burden		Non-significant results	Mixed associations
	Quantitative	Qualitative	Quantitative	Qualitative	Quantitative	Quantitative /Qualitative
Older age	El-Nagar et al., 2021	Corbett et al., 2022	Eton, Linzer, et al., 2020; Herzig et al., 2019		Aschmann et al., 2019; Eton et al., 2022; Hounkpatin et al., 2022; Hu et al., 2022; Morris et al., 2021; Siddiqui et al., 2020	van Pinxteren et al., 2023
Female					Hounkpatin et al., 2022; Hu et al., 2022; Morris et al., 2021; Siddiqui et al., 2020	Eton, Linzer, et al., 2020; van Pinxteren et al., 2023
Being married					Hounkpatin et al., 2022; Morris et al., 2021	
Lower level of education		Corbett et al., 2022; Morgan et al., 2019	Song et al., 2019		Eton et al., 2022; Hu et al., 2022	Eton, Linzer, et al., 2020; Herzig et al., 2019
Living in rural, suburb, unsafe, deprived areas	Herzig et al., 2019	Duguay et al., 2014; Hardman et al., 2021; van Pinxteren et al., 2023				
Being employed				van Pinxteren et al., 2023		Morgan et al., 2019; Ortenblad et al., 2018
Desire to maintain independence/valuing other life demands over treatment		Corbett et al., 2022; Ortenblad et al., 2018				

- For quantitative studies, we determined significance by considering a p-value threshold of 0.05 and the 95% confidence intervals reported by the authors. Multivariate regression analysis results were reported unless only univariate analysis results were available. For qualitative studies, we assessed relevance based on the authors' descriptions and pertinent quotations

- "Mixed associations" refers to situations where the impact of a contributing factor manifests in two divergent directions

- Contributing factors reported in at least two studies were included in this table. Contributing factors mentioned in single study were as follows: (1) Barriers that worsening treatment burden (TB): lower quality of life, diabetes, atrial fibrillation [42], longer duration of disease, number of healthcare needs [45], number of homecare visits [54], (2) Facilitators that reducing TB: frequency of follow-up, usual source of care: primary care [45], social network clustering [48], (3) Non-significant: self-reported life expectancy, antihypertensive treatment [46], race, years to death, cancer, depression, anxiety [37, 58], life purpose [36], duration of community centre visits, channel of consultations [45], (4) Mixed associations: network density [48]

Although sociodemographic factors such as age, sex, and marital status were frequently addressed in the 11 studies [5, 9, 32, 36, 37, 40–42, 45, 46, 51], most studies indicated the lack of a statistically significant association between these factors and treatment burden (*p*-values > 0.05 in the inferential statistics) [36, 37, 40, 41, 45, 46]. The relationships between education level and treatment burden were also inconsistent across the studies including a longitudinal study [5, 32, 36, 42, 45, 48, 52]. However, several studies consistently indicated that living in rural, suburb, or unsafe areas increased treatment burden because traveling to the clinic required more time and financial resources [9, 39, 42, 49] or posed a risk of assault or robbery [9]. Although having a job allowed people with multimorbidity to manage the financial demands of their health (e.g., medical expenses), it also posed a challenge of arranging clinic appointments with their work schedule [9, 38, 52]. Two qualitative studies described how participants’ identity and value affected their treatment burden [5, 38]. Specifically, people who desired to be independent and valued work over treatment reported higher levels of treatment burden.

#### Resources

##### Internal resources

Several studies indicated that decreased physical capacity [4, 6, 39], negative emotions (e.g., depressive symptoms) [4], and cognitive dysfunction [5] affected people's treatment burden (Table 4). These findings align with a quantitative study conducted in outpatient clinics, which revealed that half of participants experienced a high degree of treatment burden, demonstrating an association between perceived health status and treatment burden [51]. However, in Eton and colleagues’ study, where 42% and 29% of participants were diagnosed with depression and anxiety, respectively, factors such as a mental health diagnosis and the number of unhealthy physical or mental health days in the past 30 days did not consistently predict long-term trajectories of the burden of healthcare tasks [36].

**Table 4:** Contributing factors that affect treatment burden: Resources

**Internal resource**	**Worsening treatment burden**		**Reducing treatment burden**		**Non-significant results**	**Mixed associations**
	**Quantitative**	**Qualitative**	**Quantitative**	**Qualitative**	**Quantitative**	**Quantitative** **/Qualitative**
Physical, mental, cognitive capacity (or health status)			El-Nagar et al., 2021; Eton, Lee, et al., 2020; Eton, Linzer, et al., 2020	Corbett et al., 2022; Duguay et al., 2014; Fix et al., 2014; Hardman et al., 2021		Eton et al., 2022
Financial capacity			Eton, Linzer, et al., 2020; David T. Eton, Kathleen J. Yost, et al., 2017; Morris et al., 2021	Corbett et al., 2022; Hardman et al., 2021; Matima et al., 2018; Morgan et al., 2019; van Pinxteren et al., 2023	Eton et al., 2022; Hu et al., 2022	
Knowledge about overall health conditions/health literacy			El-Nagar et al., 2021; Herzig et al., 2019; Morris et al., 2021	Corbett et al., 2022; Duguay et al., 2014; Fix et al., 2014; Hardman et al., 2021; Matima et al., 2018; Ortenblad, 2018; van Pinxteren et al., 2023		Eton et al., 2022; Eton, Lee, et al., 2020; David T. Eton, Kathleen J. Yost, et al., 2017
Self-care skills, coping skills			Herzig et al., 2019; Schulze et al., 2022	Corbett et al., 2022; Fix et al., 2014; Hardman et al., 2021; Matima et al., 2018; Ortenblad, 2018; van Merode et al., 2018		Eton et al., 2022; Eton, Linzer, et al., 2020
Self-efficacy			David T. Eton, Kathleen J. Yost, et al., 2017	Corbett et al., 2022; Hardman et al., 2021; Matima et al., 2018		Eton et al., 2022
Positive attitude (e.g., sense of responsibility)				Corbett et al., 2022; Duguay et al., 2014; Matima et al., 2018; van Merode et al., 2018; van Pinxteren et al., 2023		
Faith and spirituality				Matima et al., 2018; Ortenblad et al., 2018		
**External recourse**	**Worsening treatment burden**		**Reducing treatment burden**		**Non-significant results**	**Mixed associations**
	**Quantitative**	**Qualitative**	**Quantitative**	**Qualitative**	**Quantitative**	**Quantitative** **/Qualitative**
Support from family and others (except healthcare provider)			Schreiner & Daly, 2020; Schulze et al., 2022	Corbett et al., 2022; Duguay et al., 2014; Matima et al., 2018; Morgan et al., 2019; Ortenblad, 2018; van Pinxteren et al., 2023		Eton et al., 2022
Support from healthcare provider (e.g., shared decision making)			David T. Eton, Jennifer L. Ridgeway, et al., 2017; Tinetti et al., 2019*	Corbett et al., 2022; Duguay et al., 2014; Hardman et al., 2021; Matima et al., 2018; Ortenblad, 2018; van Merode et al., 2018; van Pinxteren et al., 2023	Hu et al., 2022; McCarthy et al., 2022*	
Support from the healthcare system (e.g., multidisciplinary and coordinated care)			Hounkpatin et al., 2022; Hu et al., 2022	Corbett et al., 2022; Duguay et al., 2014; Hardman et al., 2021; Matima et al., 2018; Morgan et al., 2019; Ortenblad, 2018; van Merode et al., 2018; van Pinxteren et al., 2023		Eton, Linzer, et al., 2020; David T. Eton, Kathleen J. Yost, et al., 2017
Support from the government (e.g., pension, grant, policy)				Hardman et al., 2021; Matima et al., 2018; van Merode et al., 2018; van Pinxteren et al., 2023		

^*^ = Interventional studies

- For quantitative studies, we determined significance by considering a p-value threshold of 0.05 and the 95% confidence intervals reported by the authors. Multivariate regression analysis results were reported unless univariate analysis results were only available. For qualitative studies, we assessed relevance based on the authors' descriptions and pertinent quotations

- "Mixed associations" refers to situations where the impact of a contributing factor manifests in two divergent directions

- Contributing factors reported in at least two studies were included in this table. Contributing factors mentioned in single study were as follows: (1) Barriers that worsening treatment burden (TB): lower quality of life, diabetes, atrial fibrillation [42], longer duration of disease, number of healthcare needs [45], number of homecare visits [54], (2) Facilitators that reducing TB: frequency of follow-up, usual source of care: primary care [45], social network clustering [48], (3) Non-significant: self-reported life expectancy, antihypertensive treatment (47), race, years to death, cancer, depression, anxiety [37, 58], life purpose [36], duration of community centre visits, channel of consultations [45], (4) Mixed associations: network density [48]

Several qualitative studies highlighted that people living with multimorbidity often faced financial difficulties in performing healthcare tasks [4, 5, 8, 9, 52]. This finding is aligned with the finding that paying for healthcare costs was associated with an increase in treatment burden [41]. However, household income levels did not predict the trajectory of healthcare tasks and impact over 24 months in Eton and colleagues’ study where 55% of the participants had a household income below the country’s median [36].

Several qualitative studies found that people with multimorbidity who were knowledgeable about their health conditions and had adequate health literacy were likely to actively communicate with their healthcare providers and clearly comprehend their illness, which reduced the burden of managing their health conditions [5, 6, 8, 9, 38, 39, 49]. One study also found that people’s health literacy was associated with the burden from the trajectory of healthcare tasks, but not the burden from the impact [36]. However, their study measured health literacy with only one item, asking about their perceived difficulty understanding the provided medical information.

Self-efficacy and self-care skills including coping skills were valuable assets for lowering treatment burden [4–6, 8, 38, 42, 53, 55]. People who accepted their health tasks and maintained hope through faith and spirituality experienced lower treatment burden [5, 8, 9, 38, 39, 53].

##### External resources

People with multimorbidity who received support from family members and others reported experiencing reduced burden from healthcare tasks and the negative impacts [5, 8, 9, 38, 39, 52, 54, 55]. They noted the integral role of caregivers who could share responsibility for some of the patients’ self-care activities and life demands (e.g., household chores and financial support). Eton and colleagues found that distress from negative relations with members of the patients’ social networks (e.g., interpersonal challenges) was associated with both the trajectory of burden from healthcare tasks and the impact, while social support, in general, was unrelated to either burden from healthcare tasks or impact [36].

Many participants in six qualitative studies expressed frustration with unsupportive healthcare providers [5, 8, 9, 38, 49, 53]. Tinetti and colleagues' interventional study demonstrated that the implementation of care aligned with the priority of the people with multimorbidity via shared decision-making was effective in reducing treatment burden [43]. These findings have been further supported by other studies indicating the importance of healthcare providers' empathic attitude and provision of comprehensive information with appropriate communication skills [5, 8, 9, 31, 38, 39, 49, 53].

Positive experiences of people with multimorbidity in a primary care setting along with government support (e.g., old age pension and supportive policy) were associated with a decrease in treatment burden by reducing the financial impact [8, 9, 45, 49, 53]. In contrast, factors that frequently increase treatment burden included poor access to the healthcare system, dissatisfaction with the quality of care, and discontent and challenges with the fragmented healthcare system [5, 8, 9, 38–40, 49, 52]. One participant with multimorbidity described the struggles: "It’s not the disease that I’m fighting; it’s the healthcare system” [39].

### Health outcomes of treatment burden

The health outcomes of treatment burden were described in 11 studies (five quantitative and six qualitative studies) [5, 6, 8, 9, 12, 32, 38, 47, 50, 53, 55] (Table 5). The most commonly described health outcomes across the studies was non-adherence to self-care activities, with the main activity being medication non-adherence [5, 6, 9, 12, 32, 33, 38, 53, 55]. Non-adherence was an intentional action (e.g., ignore or modify required guidance) [5, 6, 9, 38, 53] or a non-intentional action [9], but most studies found that intentional non-adherence was prevalent. For example, Corbett and colleagues found that several participants strategically chose to deviate from or ignore recommended therapeutic regimens in order to "live their life as they wanted" [5].

**Table 5:** Health outcomes related to an increase in treatment burden

Health outcomes	Significant results		Non-significant results	Mixed associations
	Quantitative	Qualitative	Quantitative	Quantitative /Qualitative
Reduced adherence to treatment	**Medication adherence** Eton, Lee, et al., 2020; Schulze et al., 2022	**Overall adherence** Corbett et al., 2022; Fix et al., 2014; Ortenblad, 2018; van Merode et al., 2018; van Pinxteren et al., 2023		**Medication adherence** Eton, Linzer, et al., 2020; David T. Eton, Kathleen J. Yost, et al., 2017
Deterioration of health status, disease progression	**General mental and physical health status at 6 months after the baseline** Eton et al., 2019	**Disease progression** **/decreased functional status** Matima et al., 2018; Ortenblad, 2018; van Pinxteren et al., 2023	**Global health status** Chin et al., 2019	**Functional status** Chin et al., 2019
Lower quality of life	**Health-related quality of life** Chin et al., 2019; David T. Eton, Kathleen J. Yost, et al., 2017; Schulze et al., 2022			
Greater caregiver burden		**Impact on families** Ortenblad, 2018		

- The results of the quantitative studies were all documented in the table regardless of their significance

- For quantitative studies, we determined significance by considering a *p*-value threshold of 0.05 and the 95% confidence intervals reported by the authors. Multivariate regression analysis results were reported unless univariate analysis results were only available. For qualitative studies, we assessed relevance based on the authors' descriptions and pertinent quotations

- "Mixed associations" refers to situations where the impact of a contributing factor manifests in two divergent directions

The disease progression and deterioration of health status was another health outcome described in the studies [8, 9, 38, 47, 50]. Eton and colleagues showed that higher levels of treatment burden were associated with mental and physical health status six months after the baseline [47]. A relationship between treatment burden and quality of life was also found in three studies [12, 50, 55]. Caregiver burden due to healthcare tasks of people living with multimorbidity and their impacts on caregivers’ daily lives was also described in Ortenblad and colleagues’ study [38]. In their study, people living with multimorbidity reported that their family members faced the challenge of not being able to enjoy their own personal and social activities as they prioritized the health of their family member with multimorbidity.

### Instruments measuring treatment burden in multimorbidity

To evaluate the comprehensiveness of the instruments, we analyzed seven quantitative studies that reported the psychometric properties of the instruments. Three instruments and their variations were found: PETS and its variations (i.e., the brief version of PETS and PETS version 2.0) [12, 32–34]; the Chinese version of the TBQ [50], and the Chinese and German version of the MTBQ [55, 56] (see Supplementary file 4). The number of items in each instrument varied: 60 items in PETS version 2.0 [33], 15 items in the TBQ [50], and 13 items in the MTBQ [58].

Among the three versions of the PETS included in the review, the latest version of PETS version 2.0 was used to examine the contents because this latest version was more comprehensive compared to the original PETS [12, 33]. In addition, there were deleted items in the final translated versions of the MTBQ [55, 56]. In the process of cultural adaptation, translated versions of the MTBQ often excluded items that were irrelevant to local healthcare systems. For instance, in the German version of the MTBQ, the item, "Getting help from community services" was removed due to no similar service structures in Germany [55]. Therefore, to ensure a comprehensive evaluation of item content, we opted to use the original version of the MTBQ [58].

#### Comprehensiveness of the contents

Items in PETS version 2.0, the TBQ, and the MTBQ addressed both components of treatment burden (i.e., healthcare tasks and the impacts) (Table 2) [33, 50, 55, 56, 58]. However, some items in the three instruments asked about resources that exacerbated treatment burden (e.g., “problems with different healthcare providers not communicating with each other about my medical care” in PETS version 2.0) [33].

Three groups of healthcare tasks that people with multimorbidity performed were included in the three instruments: self-care activities, knowledge acquisition, and paperwork. All three instruments addressed self-care activities (e.g., medication management and health status and symptom monitoring) [33, 50, 55, 56, 58]. However, the detailed contents of the items in each instrument varied slightly. For instance, items in the TBQ and the MTBQ only addressed the burden of exercising and changing one’s diet for self-care activities [50, 55, 56, 58]. However, items in PETS version 2.0 also asked about difficulties related to using medical equipment [33].

Items asking about knowledge acquisition were found in PETS version 2.0 and the MTBQ [33, 55, 56, 58]. However, items in PETS version 2.0 asked about learning various information (e.g., healthy food, medications, and treatment plans), while the item in the MTBQ asked about obtaining information that was understandable and up-to-date. Items about paperwork were addressed in PETS version 2.0 and the TBQ [33, 50]. However, no instrument included items asking about the burden of constant prioritization between healthcare tasks and people’s personal lives or among the various healthcare tasks [5, 8, 38, 39, 52, 53].

Three types of impact from healthcare tasks on people’s lives were identified in our review: social, emotional, and financial impacts (Table 2). PETS version 2.0 and the TBQ addressed all three types of impact [33, 50], while the MTBQ included only social and financial impact [55, 56, 58]. Among the items related to social impact, being dependent on others was included in all three instruments [33, 50, 55, 56, 58]. Role/social activity limitations were only addressed in PETS version 2.0 [33]. Emotional impact that was included in PETS version 2.0 and the TBQ [33, 50] were slightly different. PETS version 2.0 asked about mental exhaustion such as anger, frustration and depression due to self-management [33], while the TBQ included one item related to how they felt about being sick (“The need for medical health care on a regular basis reminds me of my health problems”) [50]. Financial impacts were addressed in all three instruments [33, 50, 55, 56, 58], but the level of exhaustiveness and details varied slightly among the three instruments. Items in PETS version 2.0 asked about the burden of paying for medications, healthy foods, and medical expenses as well as the impact of medical costs on future plans [33].

## Discussion

We found that treatment burden consisted of burden from four healthcare tasks (i.e., self-care activities, knowledge acquisition, paperwork, ongoing prioritization) and their impacts on social, emotional, and financial lives of people with multimorbidity. In the context of multimorbidity, individual’s circumstances and available resources affected their treatment burden. We also found that items included in the existing instruments measuring treatment burden in this population did not address all the details of the components of treatment burden identified in our review.

Our review showed that people with multimorbidity felt the burden of treatment on their lives from various healthcare tasks and the impacts of the tasks. This finding is consistent with previous studies describing the conceptual framework and taxonomy of treatment burden for people with chronic conditions [18, 19]. However, our integrated map revealed two additional unique aspects of treatment burden of multimorbidity along with contributing factors and health outcomes of treatment burden. First, we identified ongoing prioritization as a healthcare task that has not been explicitly addressed in previous conceptual models or taxonomy [18, 19] or in instruments measuring treatment burden in people with multimorbidity [12, 32–34, 50, 55, 56, 58]. Although PETS version 2.0 included items asking about the role (e.g., roles in workplace and family) and social activity limitations due to healthcare tasks, ongoing prioritization was not considered a healthcare task [33]. We also found that ongoing prioritization included not only prioritizing between their healthcare tasks and people’s daily lives but also prioritizing among people’s various healthcare tasks.

Our review showed that people living with multiple chronic conditions frequently faced the additional challenge of setting day-to-day priorities and decision-making [5, 8, 38, 39, 52, 53]. This finding has also been well described in previous review papers [59, 60]. Some investigators have mentioned prioritization as a strategy to alleviate treatment burden of people living with multimorbidity [15, 61, 62]. However, in our study, we specifically identified ongoing prioritization as a distinct healthcare task based on the iterative nature of managing chronic conditions [63, 64]. Paterson and colleagues reported that people with a single disease made an average of 21 decisions related to self-care per day [65], underscoring the continuous nature of this task. For people living with multimorbidity, the act of setting priorities is an ongoing task because they frequently experience changes in disease status, which could prompt them to consider how to manage their health given their available resources and circumstances [5, 39, 49]. Yin and colleagues noted that this type of healthcare task is not always visible to others and is often unappreciated, so people living with multimorbidity may receive little assistance from others [66].

Second, our integrated map explicitly describes the role of multimorbidity in understanding the treatment burden of people living with multimorbidity. Most identified healthcare tasks performed by patients with multimorbidity in our review and other studies are comparable to those performed by patients with a single chronic condition [67, 68]. For example, people with heart failure should adhere to multiple medications for heart failure, a low sodium diet, and symptom monitoring, and they should keep their appointments with cardiologists [67]. However, when people with heart failure are diagnosed with new chronic conditions, the quantity and complexity of the tasks can significantly increase, such as difficulty interpreting changes in symptoms [69, 70]. Thus, the treatment burden of people living with multimorbidity was distinct compared to people with a single chronic condition because of the context of multimorbidity.

Our review revealed three types of impact on healthcare tasks: social, emotional, and financial, which have been consistently addressed in previous conceptual models and taxonomy of multimorbidity treatment burden [18, 19] and instruments measuring treatment burden [12, 32–34, 50, 55, 56, 58]. However, unlike previous models and taxonomy, the reciprocal relationship between healthcare tasks and the impacts is reflected in our integrated map. Given that only two studies in our review showed this interrelated relationship [36, 38], further investigation is needed to support the association between healthcare tasks and impacts for people with multimorbidity.

Studies have frequently investigated resources and included them in previous conceptual frameworks or taxonomy of the treatment burden of people with multimorbidity [14, 18, 19, 21, 71]. Knowledge about health conditions and health literacy were identified in several studies as internal resources, while support from the healthcare system (e.g., accessibility to care, multidisciplinary and coordinated care, improvement of care quality) was been frequently mentioned as external resources in our review and previous studies [15, 16, 71]. Knowing what resources are accessible to people living with multimorbidity is critical. Shippee’s Cumulative Complexity Model suggests that the treatment burden arises from an imbalance between patients’ workload and capacity, which refers to their preparedness to meet various demands [15]. Thus, to successfully decrease the treatment burden, healthcare providers should have a holistic view when helping people living with multimorbidity and comprehensively assess the burden so they do not miss any key information about people’s internal and external resources and their circumstances. In particular, improving the continuity of care can be valuable to reduce their treatment burden. Continuity of care was the most frequently reported factor for reducing treatment burden in our review.

As a health outcome of treatment burden, non-adherence to treatment emerged as the most described outcome, and intentional non-adherence was the most common. This finding highlights the importance of developing interventions to decrease treatment burden in this population. For instance, shared decision-making could serve as an effective strategy to mitigate the treatment burden associated with multimorbidity. Tinetti and colleagues conducted an intervention study on people with multimorbidity and found that discussing self-care activities and medical procedures with this group based on their life priorities was effective to decrease in treatment burden [43]. They found that the intervention led to increased medication discontinuation, decreased orders for diagnostic/laboratory tests, and fewer additional self-care activity recommendations. Our review also revealed that treatment burden amplified caregivers’ burden, which could ultimately lead to depleted social resources. However, although several studies have indicated that caregiver burden is an important factor affecting health outcomes of people with chronic illness [25, 72, 73], only one study in our review reported this relationship [38].

Our review found that three measures of treatment burden adequately addressed the majority of the specific components of treatment burden. However, none of the three measures included items about ongoing prioritization. Regarding impacts, the TBQ included one item for each of the three categories of impacts (i.e., social, emotional, and financial) [74] and the MTBQ included no item about emotional impact [58]. Although items in PETS version 2.0 addressed treatment burden in great detail, the measure is lengthy with 60 items, and some of the items assessed components other than treatment burden (e.g., resources) [33]. Both the TBQ and the MTBQ contained items indicating resources, which is not a component of treatment burden based on the definition of treatment burden (i.e., the burden from performing healthcare tasks and the impact of those tasks on the well-being of people living with multimorbidity) [58, 74]. Thus, the measures of multimorbidity treatment burden need further improvement by considering the contents and applicability in clinical settings.

### Limitations

There are limitations to be noted in our review. The participants of the included studies were mostly from Western countries and were older people, which limits the generalizability of our findings to the population with multiple chronic conditions. Excluding non-English articles also limited the comprehensiveness of our findings. Most of the studies included in the review used a medical records review method to collect data on chronic conditions. Although a medical records review is considered the gold standard, self-reported chronic conditions may be more realistic. People with multimorbidity may feel burdened by healthcare tasks from the chronic conditions that they believe they have, rather than those they actually have. Thus, it is possible that studies included in our review understated the relationship between the context of multimorbidity and treatment burden.

## Conclusion

We developed an integrated map of treatment burden illustrating the dynamic relationships among treatment burden, the multimorbidity context, individual’s circumstances and available resources, and health outcomes. Our findings can help scholars and medical professionals comprehensively understand the treatment burden experienced by people living with multimorbidity and the unique features of their treatment burden. The findings can also help professionals develop person-centered interventions considering individuals’ available resources given their circumstances and the context of multimorbidity. However, more research is needed to support and refine our integrated map. We also found that existing instruments measuring multimorbidity treatment burden often overlooked certain aspects, such as ongoing prioritization, which is particularly relevant for people living with multimorbidity. Further work is also needed to develop instruments that overcome the weaknesses of the current instruments.

## Supplementary Information

Supplementary file 1.

Supplementary file 2.

Supplementary file 3.

Supplementary file 4.

## Data Availability

The datasets supporting the conclusions of this article are included within the article and its Supplementary files.

## Abbreviations

MMAT: The Mixed Methods Appraisal Tool
MTBQ: Multimorbidity Treatment Burden Questionnaire
PETS: Patient Experience with Treatment burden and Self-management
TBQ: Treatment Burden Questionnaire
