# Supplementary material for: Treatment burden in multimorbidity: an integrative review
Source: BMC Prim Care. 2024 Sep 28;25:352. doi: 10.1186/s12875-024-02586-z (PMC11438421; doi:10.1186/s12875-024-02586-z)
Supplement: Supplementary file 1 [file 12875_2024_2586_MOESM1_ESM.docx]

**[Supplementary File 1] Ovid MEDLINE® Search queries**

1 exp Comorbidity/

2 exp chronic disease/

3 (multimorbid* or multi-morbid* or comorbid* or co-morbid*).ab,ti.

4 (chronic* adj3 (disease* or ill* or care or condition* or disorder* or health* or symptom*)).ab,ti.

5 (multidisease* or multi disease* or (multiple adj (ill* or disease* or condition* or disorder*))).ab,ti.

6 1 or 2 or 3 or 4 or 5

7 ((treat* or therap* or patient*) adj3 (burden* or workload*)).ab,ti.

8 ((lifestyle* or administrative* or healthcare* or time* or travel* or procedure* or financial* or cost* or expense*) adj3 (burden* or workload*)).ab,ti.

9 ((drug* or interven* or medicat* or medicin*) adj3 (burden* or workload*)).ab,ti.

10 ((selfcare or self-care or selfmanagement or self-management or selfmonitoring or self-monitoring) adj3 (burden* or workload*)).ab,ti.

11 ((disease* or ill*) adj3 management adj3 (burden* or workload*)).ab,ti.

12 7 or 8 or 9 or 10 or 11

13 6 and 12

14 limit 13 to (english language and yr="1860 - 2022" and "all adult (19 plus years)" and journal article
