# Supplementary material for: Treatment burden in multimorbidity: an integrative review
Source: BMC Prim Care. 2024 Sep 28;25:352. doi: 10.1186/s12875-024-02586-z (PMC11438421; doi:10.1186/s12875-024-02586-z)
Supplement: Supplementary file 2 [file 12875_2024_2586_MOESM2_ESM.docx]

**[Supplementary File 2]**

**Quality evaluation of included studies using the Mixed Methods Appraisal Tool, 2018 version**

| **Study reference** | **Qualitative studies** | | | | | **Methodological quality criteria** |
| --- | --- | --- | --- | --- | --- | --- |
|  | **1.1** | **1.2** | **1.3** | **1.4** | **1.5** | 1.1. Is the qualitative approach appropriate to answer the research question?  1.2. Are the qualitative data collection methods adequate to address the research question?  1.3. Are the findings adequately derived from the data?  1.4. Is the interpretation of results sufficiently substantiated by data?  1.5. Is there coherence between qualitative data sources, collection, analysis and interpretation? |
| 1. Van Pinxteren (2023) | Y | Y | Y | Y | Y |  |
| 1. Corbett (2022) | Y | Y | Y | Y | Y |  |
| 1. Hardman (2021) | Y | Y | Y | Y | Y |  |
| 1. Morgan (2019) | Y | Y | Y | Y | Y |  |
| 1. Matima (2018) | Y | Y | Y | Y | Y |  |
| 1. Ørtenblad (2018) | Y | Y | Y | Y | Y |  |
| 1. Van Merode (2018) | Y | Y | Y | Y | Y |  |
| 1. Duguay (2014) | Y | Y | Y | Y | Y |  |
| 1. Fix (2014) | Y | Y | Y | Y | Y |  |
|  | **Quantitative descriptive Studies** | | | | | 4.1. Is the sampling strategy relevant to address the research question?  4.2. Is the sample representative of the target population?  4.3. Are the measurements appropriate?  4.4. Is the risk of nonresponse bias low?  4.5. Is the statistical analysis appropriate to answer the research question? |
|  | **4.1** | **4.2** | **4.3** | **4.4** | **4.5** |  |
| 1. Hounkpatin (2022) | Y | Y | Y | C | Y |  |
| 1. Eton (2022) | Y | N | Y | Y | Y |  |
| 1. El-Nagar (2021) | Y | C | Y | C | N |  |
| 1. Morris (2021) | Y | C | Y | Y | Y |  |
| 1. Siddiqui (2021) | Y | N | Y | Y | Y |  |
| 1. Hu (2021) | Y | C | Y | Y | Y |  |
| 1. Schreiner (2020) | N | C | N | C | Y |  |
| 1. Aschmann (2019) | Y | C | Y | Y | Y |  |
| 1. Herzig (2019) | Y | Y | Y | C | Y |  |
| 1. Eton (2019) | Y | C | Y | C | Y |  |
| 1. Song (2019) | N | C | Y | C | Y |  |
| 1. Eton (2017) | Y | Y | Y | C | Y |  |
|  | **Quantitative randomized controlled trials** | | | | | 2.1. Is randomization appropriately performed?  2.2. Are the groups comparable at baseline?  2.3. Are there complete outcome data?  2.4. Are outcome assessors blinded to the intervention provided?  2.5 Did the participants adhere to the assigned intervention? |
|  | **2.1** | **2.2** | **2.3** | **2.4** | **2.5** |  |
| 1. McCarthy (2022) | Y | Y | Y | Y | N |  |
|  | **Quantitative non-randomized studies** | | | | | 3.1. Are the participants representative of the target population?  3.2. Are measurements appropriate regarding both the outcome and intervention (or exposure)?  3.3. Are there complete outcome data?  3.4. Are the confounders accounted for in the design and analysis?  3.5. During the study period, is the intervention administered (or exposure occurred) as intended? |
|  | **3.1** | **3.2** | **3.3** | **3.4** | **3.5** |  |
| 1. Tinetti (2019) | Y | Y | Y | Y | C |  |
|  | **Quantitative descriptive Studies** | | | | | 4.1. Is the sampling strategy relevant to address the research question?  4.2. Is the sample representative of the target population?  4.3. Are the measurements appropriate?  4.4. Is the risk of nonresponse bias low?  4.5. Is the statistical analysis appropriate to answer the research question? |
|  | **4.1** | **4.2** | **4.3** | **4.4** | **4.5** |  |
| 1. Schulze (2022) | Y | C | Y | Y | Y |  |
| 1. Lee (2021) | Y | N | Y | Y | Y |  |
| 1. Dou (2020) | N | Y | Y | C | Y |  |
| 1. Eton (2020) | Y | N | Y | Y | Y |  |
| 1. Eton (2020) | Y | C | Y | Y | Y |  |
| 1. Chin (2019) | Y | Y | Y | C | Y |  |
| 1. Eton (2017) | Y | Y | Y | C | Y |  |

Note. Y: yes, N: no, C: can’t tell. The study numbers #11, 25, 27 used the identical dataset. This quality assessment was conducted using the Mixed Methods Appraisal Tool (MMAT), version 2018 (1).

1. Hong QN, Fàbregues S, Bartlett G, Boardman F, Cargo M, Dagenais P, et al. The Mixed Methods Appraisal Tool (MMAT) version 2018 for information professionals and researchers. Education for information. 2018;34(4):285-91.
