# Supplementary material for: Treatment burden in multimorbidity: an integrative review
Source: BMC Prim Care. 2024 Sep 28;25:352. doi: 10.1186/s12875-024-02586-z (PMC11438421; doi:10.1186/s12875-024-02586-z)
Supplement: Supplementary file 4 [file 12875_2024_2586_MOESM4_ESM.docx]

**[Supplementary File 4] Characteristics of instruments**

| **Instruments** | | **Recall period** | **Number of items** | **Response options** | **Range of scores*** | **Mean time to administer** | **Language** |
| --- | --- | --- | --- | --- | --- | --- | --- |
| **PETS** | PETS (1) | Past 4 weeks | 48 items: 9 dimensions and 2 medication bother items | 4- or 5-point ordered categorical response | 0-100 | Not reported | English |
|  | PETS version. 2.0 (2, 3) | Past 4 weeks | 60 items: 10 dimensions and 2 medication bother items | 4- or 5-point ordered categorical response | 0-100 | Not reported | English |
|  | PETS short version (4) | Past 4 weeks | 32 items: 10 dimensions and 1 medication bother item | 4- or 5-point ordered categorical response | 0-100 | Not reported | English |
| **TBQ** | C-TBQ (5) | In general | 15 items | 10-point Likert | 0-150 | 10.2 minutes | Chinese |
| **MTBQ§** | MTBQ German version (6) | In general | 11 items | 5-point Likert | 0-100 | 4 minutes | German |
|  | C-MTBQ  (7) | In general | 12 items | 5-point Likert | 0-100 | 5 minutes | Chinese |

Note. MTBQ: Multimorbidity Treatment Burden, PETS: Patient Experience with Treatment and Self-Management, TBQ: Treatment Burden Questionnaire

* Higher scores of all instruments indicate a greater level of treatment burden.

§ Although the initial MTBQ consisted of 13 items, the final versions of the translated MTBQ excluded one or two items.
